# Short-Term Pharmacokinetics of Ropivacaine in Arterial Plasma During Erector Spinae Plane Block in Thoracic and Cardiac Surgery

**DOI:** 10.3390/pharmaceutics18081020

**Published:** 2026-08-17

**Authors:** Amedeo De Nicolò, Alessandra Manca, Edoardo Ceraolo, Giulio Luca Rosboch, Antonio Toscano, Luca Neitzert, Eleonora Balzani, Jessica Cusato, Alice Palermiti, Giorgia Giuseppina Montrucchio, Antonio D’Avolio

**Affiliations:** 1 Laboratory of Clinical Pharmacology and Pharmacogenetics, Department of Medical Sciences, University of Turin, 10149, Turin, Italy; alessandra.manca@unito.it (A.M.); jessica.cusato@unito.it (J.C.); alice.palermiti@unito.it (A.P.);; 2 Department of Anaesthesia, Critical Care and Emergency, “Città Della Salute e Della Scienza” Hospital, 10129 Turin, Italy; edoceraolo@gmail.com (E.C.); giuliorosboch@gmail.com (G.L.R.); atoscano@cittadellasalute.to.it (A.T.); giorgiagiuseppina.montrucchio@unito.it (G.G.M.); 3 Department of Surgical Sciences, University of Turin, 10126 Turin, Italy; luca.neitzert@unito.it (L.N.); eleonora.balzani@unito.it (E.B.)

**Keywords:** anesthetics, local, drug-related side effects and adverse reactions, pharmacokinetics, nerve block, thoracic surgical procedures

## Abstract

**Background/Objectives**: Local anesthetics (LAs) are used in a variety of different contexts, from loco-regional anesthesia to analgesia. In cardiothoracic surgery, fascial plane blocks are deserving of attention, including erector spinae plane block (ESPB). In this context, ropivacaine is convenient, due to its peculiar pharmacokinetic/pharmacodynamic properties. Nevertheless, LAs can still cause systemic toxicity (LAST), due to erroneous injection and/or variable systemic adsorption/distribution. This interindividual pharmacokinetic variability can be intensified by the tendency to use a fixed ropivacaine dose in ESPB. The aims of this work were investigating systemic exposure to ropivacaine during ESPB in the context of cardiac and thoracic surgery, comparing it to the literature-reported maximum tolerated concentrations, and identifying potential predictors of exposure. **Methods**: Patients receiving ultrasound-guided injection of 40 mL of ropivacaine 0.375% solution for ESPB were enrolled. Arterial blood was sampled at 5, 15, 30, 45, 60, 120, and 180 min after the injection, and total and free concentrations of ropivacaine were determined by means of LC-MS/MS analysis of arterial plasma. **Results**: Concentrations showed wide variability, particularly during the first hour post-dose. Significant differences were observed between patients undergoing cardiac and thoracic surgery, with the latter showing higher concentrations, potentially above the cutoff values predictive of LAST. Pharmacokinetic differences were mainly explained by anthropometric (body weight and BSA) and clinical/hemodynamic (NYHA score) characteristics. **Conclusions**: This study shows that about 5% of patients could reach arterial plasma concentrations of ropivacaine above the cutoff level for LAST after ESPB with a 150 mg dose. Considering patients’ weight could be beneficial to maintain exposure below the toxicity cutoff.

## 1. Introduction

In recent years, loco-regional anesthesia (LRA) techniques have constantly been improving, including with the use of ultrasound-assisted techniques, leading to the development of safer and more effective techniques such as muscular plane blocks (MPBs) [1,2,3]. These techniques consist of the injection of relatively high volumes of solutions containing low concentrations of local anesthetics (LAs) between muscular planes, allowing the LA to diffuse to nearby nervous structures.

Among these, erector spinae plane block (ESPB) is mainly used in thoracic and cardiac surgery, due to its high effectiveness, safety, and simpler ability to identify, by means of ultrasound imaging, the fascial plane where the LA should be delivered [1,3].

Different LAs are currently being used for ESPB, including bupivacaine, levobupivacaine, lidocaine, and ropivacaine (RPV), the latter of which is deserving of attention since it presents reduced lipophilicity and high potency. These properties provide strong local effects with slower systemic absorption and minor penetration into the tissues, leading to better analgesic properties and weaker effects on motor and sensitive fibers [4]. Nevertheless, despite the adoption of safer techniques and more convenient pharmacokinetic/pharmacodynamic (PK/PD) properties, some cases of LA-related systemic toxicity (LAST) continue to occur [5,6], sometimes due to erroneous administration (accidental endovenous injection) or due to the peculiarities of each patient, such as a low volume of distribution, high vascularity and perfusion, or low levels of plasma proteins [7]. The occurrence of LAST is fortunately uncommon, usually transient/reversible and manageable, and can be observed soon after the administration of LAs, typically with neurological (e.g., seizure) or sometimes cardiac manifestations; however, some fatal cases have been described [6,7,8,9,10,11,12,13]. RPV is highly bound to α1-acid glycoprotein and secondarily to albumin, and significantly higher free concentrations can be observed in patients who have low concentrations of these proteins [7,10,11,14,15,16].

To complicate this issue, some relevant artero-venous differences in RPV concentrations have been described, with arterial concentrations being higher and thus being expected to have a stronger impact on LAST [8].

Finally, unlike drugs used for a systemic effect, RPV is typically administered at a fixed standard dose (concentration and volume) in the context of ESPB protocols to obtain high local effect within the maximum tolerated dose [17]. Recent works suggested the injection of 40 mL of 0.375% RPV solution for monolateral (or 20 mL for bilateral) ESPB to be effective and safe [18,19,20]. However, fixed doses are often associated with high inter-individual systemic PK variability.

Therefore, since the PK/PD characteristics of RPV in ESPB in real life are poorly explored, including the interindividual variability in peak concentration (C_max_) and time to peak concentration (T_max_), which are expected to be the parameters with the greatest impact on risk of LAST, this work sought to (1) describe RPV systemic exposure and the proportion of cases above the literature-derived MTCs [8], (2) study concentration differences in different wards and in patients with different characteristics, related to their clinical conditions, and (3) test the correlation (and predictive power) between patients’ characteristics and RPV systemic exposure.

## 2. Patients and Methods

### 2.1. Study Design and Power Analysis

This study was designed to describe the pharmacokinetics of RPV in a cohort of patients undergoing ESPB in thoracic and cardiac surgery with a fixed dose and volume of the drug, following the same procedure. The primary endpoint was the description of systemic RPV concentrations during the surgical intervention (about 3 h) in relation to the literature-described maximum tolerated concentrations (MTCs). Since the primary endpoint was mainly descriptive and exploratory, sample size calculation was performed to identify, through linear regression analysis, up to 2 independent predictors of RPV concentration with 90% power, assuming a medium-large size of the effect of each single predictor and a loss margin of 10% (patients excluded due to possible protocol deviations). This power analysis was repeated to confirm the ability, based on two groups of 17 participants in each ward, to identify inter-ward differences of more than 20% in PK parameters, assuming a 20% relative standard deviation for the interindividual variability, and 90% power, allowing for 10% loss. These calculations determined the ideal total sample size to be 34, divided equally into 2 groups of 17 participants per ward.

As a reference for arterial plasma maximum tolerated concentrations (MTCs), values of 4300 ng/mL and 560 ng/mL were used, as determined by Knudsen et al. after intravenous infusion [8].

### 2.2. Patients’ Enrolment and Sample Withdrawal

Participants undergoing elective thoracic or cardiac surgery who received preoperative ESPB and provided written informed consent were included in this study. The exclusion criteria were as follows: neuropathies of the chest, neuropsychiatric disorders, severe renal or hepatic insufficiency, alterations of hepatic enzymes, pregnancy, hypoalbuminemia, or ICU admission with sedation for >24 h. The protocol was approved by the Ethical Committee “A.O.U. Citta’ della Salute e della Scienza di Torino—A.O. Ordine Mauriziano—A.S.L. Città di Torino”, protocol “PRAST” number 0040,318 on 7 April 2022. All patients received unilateral locoregional anesthesia. The administration of a fixed dose of 40 mL of RPV 0.375% (150 mg) was performed with an ultrasound guide between muscular planes. All surgical procedures were then carried out under general anesthesia. Induction of general anesthesia was achieved with intravenous sedatives (propofol, midazolam, or etomidate) and opiates (fentanyl or sufentanil). Neuromuscular blockade was achieved with IV bolus and continuous infusion of cisatracurium or rocuronium. In the cardiac surgery subgroup, all surgical procedures were performed through minimally invasive thoracotomy. Cardiopulmonary bypass was established after induction of general anesthesia, and myocardial protection was achieved using cardioplegic arrest. Systemic hypothermia was applied during cardiopulmonary bypass according to standard institutional protocols.

Patients’ body temperature and vital parameters were continuously monitored, alongside continuous arterial blood gas analysis (BGA), as per common clinical practice.

After the injection of RPV, arterial blood withdrawal was performed with lithium/heparin tubes at 5, 15, 30, 60, 120 and 180 min for the description of its early arterial pharmacokinetics. These samples were centrifuged at 1400× *g* to obtain arterial blood plasma, stored at −80 °C, and were then sent to the Laboratory of Clinical Pharmacology and Pharmacogenetics of the University of Turin for analysis.

Postoperatively, all patients received multimodal analgesia with IV administration of acetaminophen and NSAIDs. In case of inadequate pain control, patients received rescue opioid administration as needed.

### 2.3. Pharmacokinetic Analysis

The analysis of RPV plasma concentrations was performed by means of a validated ultra-high performance liquid chromatography coupled with tandem mass spectrometry (UHPLC-MS/MS) method for the quantification of total and free concentrations [21]. Briefly, 50 µL of plasma was added to stable-isotope-linked RPV as an internal standard (IS) and subjected to protein precipitation with a mixture of acetonitrile and methanol (50:50 vol:vol). Then, each sample was centrifuged and 100 µL of the supernatant was diluted with 400 µL of pure water. These extracts were directly injected into the LX-50 QSight 220 ^®^ (Perkin Elmer, Shelton, CT, USA) UHPLC-MS/MS system for the analysis of total RPV concentrations. Meanwhile, for the analysis of free RPV, 500 µL of plasma was kept at 37 °C for 10 min before undergoing centrifugation at 37 °C for 25 min at 2000× *g* in Centrifree ^®^ tubes (Merk Millipore, Burlington, MA, USA) to isolate free RPV and discard the protein bound fraction. One hundred microliters of the ultrafiltrate was spiked with IS, added to 100 µL of acetonitrile/methanol 50:50 (vol:vol), and diluted with 400 µL of water. Calibration standards and quality controls for the free RPV concentration were prepared with plasma ultrafiltrate to increase the robustness of the method in terms of recovery and matrix effect. The accuracy and precision of the method for total and free ropivacaine adhered to EMA ICH guidelines, with both the bias (measure of inaccuracy) and coefficients of variation (measure of imprecision) being lower than 10%. The LLOQ for the total RPV assay was 46.8 ng/mL (LOD = 20 ng/mL), while the LLOQ for free RPV concentration was 7.8 ng/mL (LOD 3.9 ng/mL). A detailed description of the method and analytical performance is provided in the methodological paper [21].

### 2.4. Statistical Analysis

Areas under the concentration–time curves and all the pharmacokinetic parameters were estimated by means of noncompartmental analysis (NCA) using Phoenix WinNonlin ^®^ software version 8.5 (Certara, Cranbury, NJ, USA). The comparison between study groups (thoracic vs. cardiac surgery) was performed by comparing 95% confidence intervals of the geomean, in order reduce the possible impact of outliers. Normal distribution was tested via the Shapiro–Wilk test. Correlations or associations between anthropometric/demographic characteristics and PK parameters were tested using the Pearson correlation test or Student *t*-test for continuous or categorical variables, respectively, using SPSS software version 30.0 (IBM, Armonk, NY, USA).

## 3. Results

### 3.1. Patients’ Baseline Characteristics

Thirty-four patients, seventeen per ward, were enrolled in this study, with no patient falling within the exclusion criteria. The characteristics of all patients at baseline are summarized in Table 1, stratified by surgical ward. Briefly, patients were predominantly female, with a median age of 70 y.o. (IQR 60–74), body weight of 67 kg (IQR 54–77), and eGFR 89 mL/min/1.73 m^2^ (IQR 65–98).

Statistically significant differences in total body weight and BSA were observed between wards, with cardiac surgery patients showing slightly higher values. Similarly, cardiac surgery patients had slightly lower eGFR, with borderline significance.

### 3.2. Effectiveness and Safety Data

All patients achieved satisfactory postoperative analgesia following unilateral locoregional anesthesia. No hemorrhagic or infectious complications related to the locoregional technique were observed. No signs or symptoms suggestive of local anesthetic systemic toxicity (LAST) were observed either immediately after drug administration or upon patient awakening in the postoperative period. Overall, the locoregional approach was well tolerated.

### 3.3. General Pharmacokinetic Results

RPV concentrations were measurable at all considered time points.

**Figure 1:**
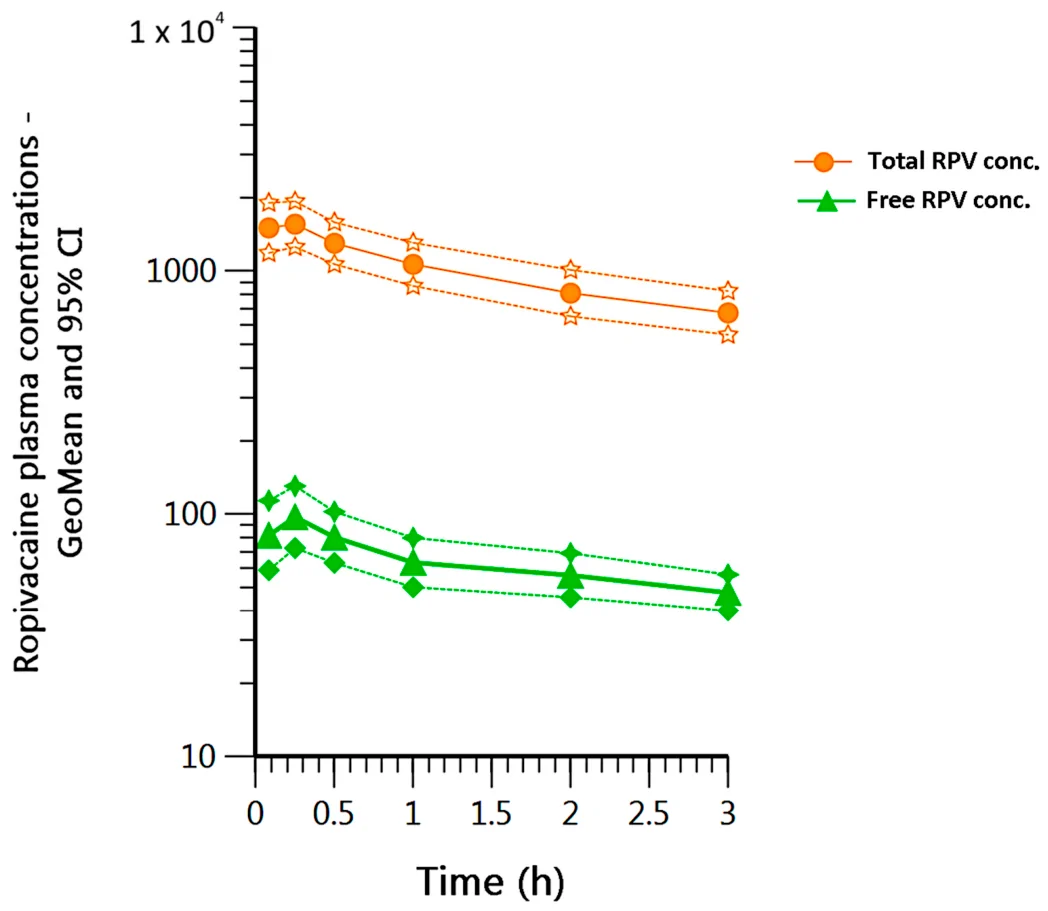
Geomean and 90% confidence intervals of the geomean of total (orange) and free (green) ropivacaine concentrations during the surgical interventions across the two surgical wards (17 participants per ward, total number = 34).

The median T_max_ was 15 min (range 5–120 min), with a geometric mean RPV C_max_ of 1558 ng/mL (CI_90_ 1256–1934) and 97.3 ng/mL (CI_90_ 72.6–130.5) for total and free RPV concentrations, respectively. The mean concentration–time curves for total and free RPV concentrations are depicted in Figure 1, while the total and free RPV PK parameters are reported in Table 2. The mean half-life was 3.0 (range 1.1–13.7) and 3.8 (range 0.8–12.8) h for total and free RPV concentrations, respectively, as can be noted by the comparable slopes in Figure 1. According to this, the geomean percentage of free RPV was 6.7% (CI_90_ 6.0–7.6) at the peak and 7.1% (CI_90_ 5.9–8.4) 3 h after administration.

### 3.4. Between Wards Pharmacokinetic Variability

**Figure 2:**
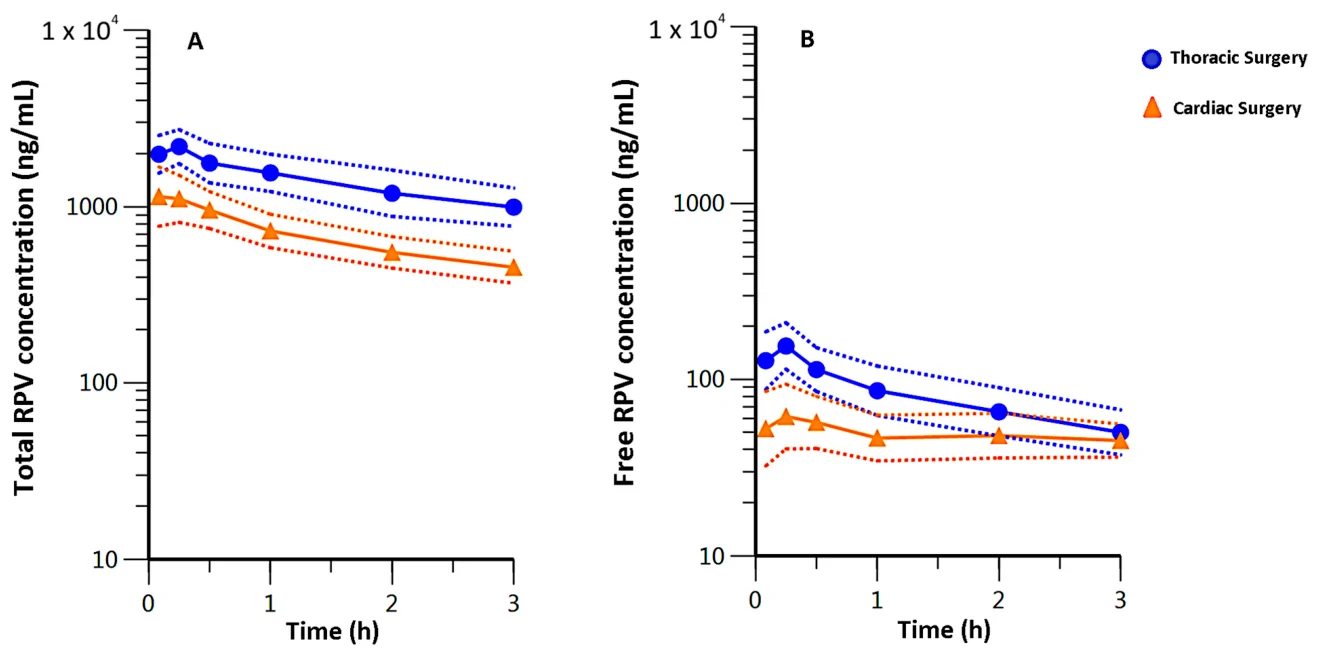
Geomean and 90% confidence intervals of the geomean (dotted lines) of total (Panel (**A**)) and free (Panel (**B**)) ropivacaine concentrations in the thoracic surgery (blue) and cardiac surgery (orange) wards (17 participants per ward, total number = 34).

Total and free RPV concentrations were observed to vary significantly between wards, as summarized in Table 2 and depicted in Figure 2. Patients from the thoracic surgery ward reached a higher AUC, a slightly anticipated T_max_ (for free concentration), and a higher C_max_ for both total and free concentrations. The half-lives for total RPV concentrations were highly comparable between wards, with a geomean value of 2.9 (range 1.1–13.4) vs. 3.1 h (range 1.2–13.6).

The apparent half-life differed significantly for the free concentrations, with the patients from cardiac surgery showing a slower reduction in the free fraction over time compared to thoracic surgery, with geomean values of 4.1 h (range 0.8–13.4) vs. 2.2 h (range 1.2–4.4), respectively.

### 3.5. Impact of Patients’ Characteristics on RPV PK

Via Pearson correlation tests, patients’ BSA and total body weight were found to be significantly correlated with total and free RPV C_max_, C_min_, and AUC_0–3,_ explaining most of the inter-ward variability in RPV PK. Correlation coefficients and *p* values of the factors which showed at least a significant correlation with PK parameters are reported in Table 3.

Specifically concerning the free RPV T_max_ and the free RPV half-life, these were found to be significantly and positively correlated with the NYHA score, suggesting some impact of hemodynamics on adsorption and distribution phases. This was reflected in the delayed free RPV T_max_ and longer free RPV half-life observed for patients from the cardiac surgery ward, as depicted in Figure 2.

### 3.6. Theoretical PK/PD Impact

**Figure 3:**
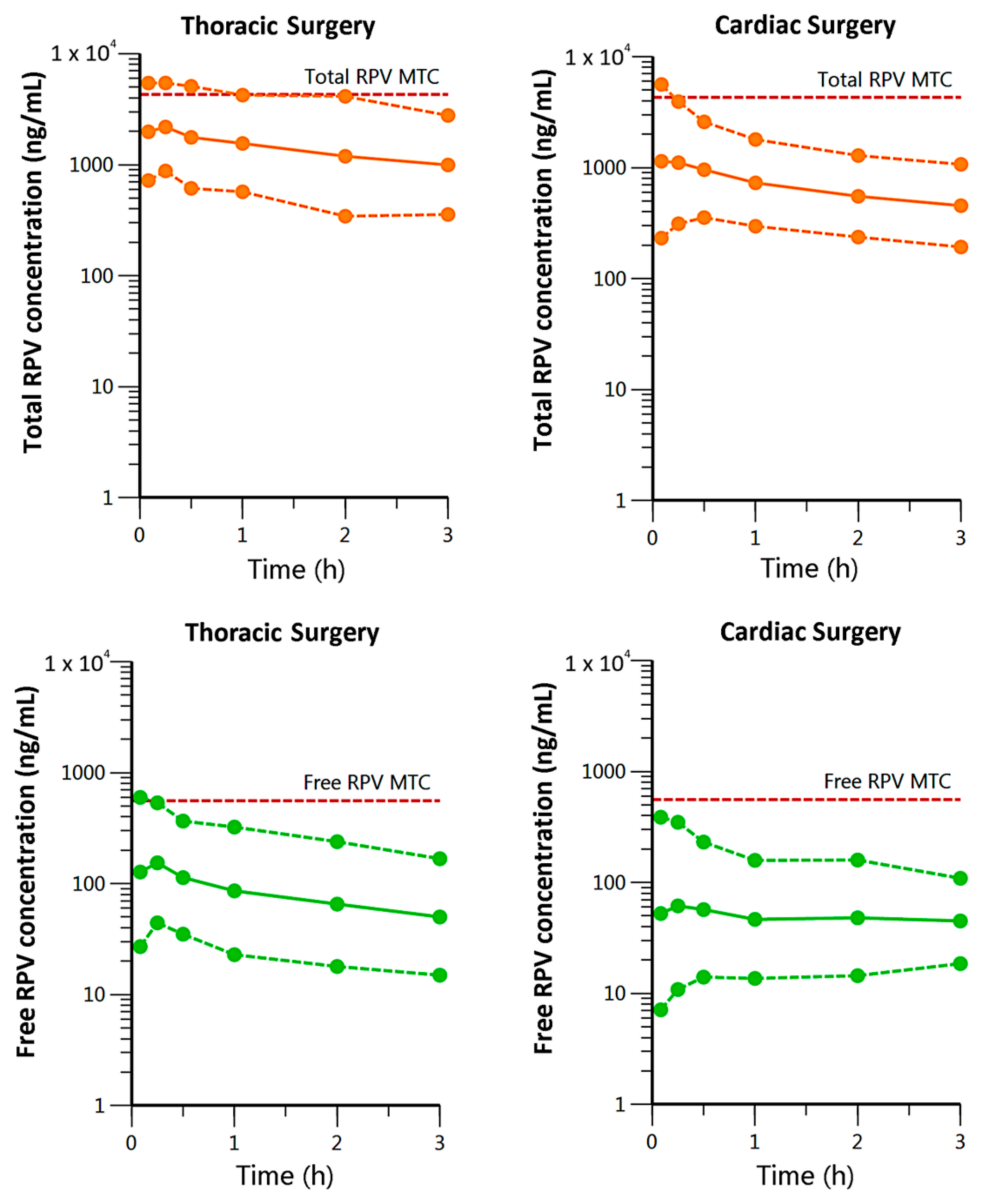
Summary depiction of the total (upper panels) and free (lower panels) ropivacaine concentrations in relation to the literature-derived maximum tolerable concentrations, depicted as horizontal red dashed lines. Solid lines represent the trends in geomean concentrations; dashed lines represents the 90% confidence interval of the general drug concentrations at each time-point. Total MTC = 4300 ng/mL; Free MTC = 560 ng/mL.

To evaluate the potential for LAST, the observed total and free RPV concentrations were compared with literature-derived suggested cutoff concentrations to predict toxicity [8]. As illustrated in Figure 3, only the upper bound of the 95% confidence interval for total RPV concentrations—and for free RPV concentrations in the thoracic surgery subgroup—approached or slightly exceeded the suggested toxicity thresholds at C_max_. However, the full confidence intervals for both total and free RPV concentrations remained below these thresholds from 1 h post-administration onwards. Notably, no clinical signs of LAST were observed in this cohort.

Finally, using a linear regression analysis, the upper boundary of the 90% confidence interval for the predicted total and free RPV concentrations exceeded the MTC of 4300 ng/mL in case of a BSA lower than 1.4, a total body weight of below 46 kg, and/or a dose/weight ratio higher than 3.2 mg/kg. Linear regression statistics and concentration prediction graphs are reported in the Supplementary Materials (Supplementary Table S1 and Figures S1–S3).

## 4. Discussion

In this work, the total and free RPV concentrations have been evaluated in the context of the ESPB technique in order to evaluate the possible risk for LAST in this common clinical practice in loco-regional anesthesia. These concentrations were lower than the suggested MTCs in arterial blood in all but one patient; however, from a statistical point of view, the 95% confidence interval exceeded these levels, suggesting that about 5% of the general population could potentially experience LAST within the first hour after the ESPB. The observed free RPV concentrations were very similar to the ones previously reported in the literature and remained stable during the observation period [11,15,16,22]. Moreover, the free RPV concentration followed the total concentration in a rather proportional manner, and the time range for potential toxicity was the same, as depicted in Figure 3. Striking evidence indicated the significant differences in concentrations between surgical wards, which were characterized by different C_max_, T_max_ and AUC_0_**_–_**_3_, but rather similar half-lives, particularly for total RPV. The parallel decrease in plasma concentrations, as reported in Figure 2, indicates that most of the PK difference is sustained by higher bioavailability and a faster absorption phase in patients from the thoracic surgery ward, as well as lower volumes of distribution, while the elimination rate is the same. No gender differences in RPV concentrations were observed, while both total and free RPV concentrations were found to be inversely correlated with total body weight and BSA, explaining some of the variability in the C_max_, C_last_ and AUC among patients. The total dose of RPV in this ESPB protocol (150 mg) was fixed for all patients, leading to different dose/weight ratios. In turn, significant differences were observed in total body weight and BSA between wards, and these differences were reflected in higher weight-adjusted dose in thoracic surgery patients, thus explaining most of the inter-ward differences. On the other hand, the T_max_ and half-life of free RPV appeared to be significantly correlated with the NYHA score, indicating the role played by patients’ hemodynamics in RPV distribution in the first hours after the injection [23].

Some limitations of this study should be acknowledged. Variables directly influencing muscular perfusion, such as cardiac output and hypothermia, were not systematically recorded; while this limits the ability to fully explore the mechanisms underlying inter-individual differences in systemic pharmacokinetics, it reflects the inherent constraints of data collection in this type of clinical cohort. Additionally, postoperative sedation following ESPB may have reduced the likelihood of detecting early neurological manifestations of local anesthetic systemic toxicity (LAST), which are typically the most common and earliest indicators of systemic exposure. Finally, analyses were conducted exclusively in arterial plasma, without a corresponding venous assessment, precluding characterization of the arteriovenous gradient. Despite its limitations, this study provides the first characterization of total and free RPV concentrations in a real-world clinical setting following ESPB, applying a standardized protocol across different surgical populations. The findings suggest that systemic pharmacokinetics may vary across clinical contexts, mainly according to differences related to body weight and NYHA score. Specifically, this study suggests that patients with a lower body weight (mainly under 45 kg) have a higher probability of reaching potentially concerning systemic concentrations.

However, considering the limited sample size and the absence of events of LAST in this cohort, it is difficult to predict the clinical relevance of this finding, meaning that the adoption of potential strategies to reduce the systemic exposure in low body weight patients, such as dose reduction (RPV concentration below 0.375% [2,24] or reduction in the volume) or the use of epinephrine as an adjuvant [22,25], should be considered with caution.

These results underscore the need for further real-world investigations to confirm and extend these observations, including in other interfascial plane blocks and across diverse patient populations, to better elucidate the determinants of local anesthetics’ disposition and their real clinical impact.

## Figures and Tables

**Table 1:** Summary of participants’ characteristics. IQR = interquartile range. ASA = NYHA = New York Heart Association Score.

	Overall Median (IQR or %); *n* = 34	Cardiac Surgery Median (IQR or %); *n* = 17	Thoracic Surgery Median (IQR or %); *n* = 17	Sign. Difference (*p* Value)
Age (y.o.)	70.5 (59.8–74.0)	71.0 (53.5–75.5)	64.0 (61.5–73.5)	0.290
Sex	F: 19 (55.9%) M: 15 (44.1%)	F: 9 (52.9%) M: 8 (47.1%)	F: 10 (58.8%) M: 7 (41.2%)	0.730
Total Body Weight (Kg)	66.5 (54.0–77.3)	70.0 (59.0–86.0)	59.0 (49.0–70)	0.018
Lean Body Weight (Kg)	48.6 (40.0–56.0)	49.0 (44.4–63.3)	41.8 (38.6–54.2)	0.079
Height (m)	1.64 (1.57–1.72)	1.67 (1.60–1.76)	1.58 (1.56–1.71)	0.150
BMI (kg/m^2^)	23.7 (20.6–26.5)	24.0 (22.1–29.5)	22.3 (19.8–25.0)	0.092
BSA (m^2^)	1.74 (1.54–1.88)	1.83 (1.63–2.04)	1.63 (1.45–1.82)	0.016
eGFR (mL/min/1.73 m^2^)	86.1 (65.5–98.0)	81.9 (60.2–86.1)	93.0 (77.0–104.5)	0.065
ASA Score	1: 1 (2.9%) 2: 7 (20.6%) 3: 24 (70.6%) 4: 2 (5.9%)	1: None 2: 1 (5.9%) 3: 14 (82.4%) 4: 2 (11.8%)	1: 1 (5.9%) 2: 6 (35.3%) 3: 10 (58.8%) 4: None	0.065
NYHA Score	1: 13 (38.2%) 2: 16 (47.1%) 3: 5 (14.7%)	1: 1 (5.6%) 2: 12 (70.6%) 3: 4 (23.5%)	1: 12 (70.6%) 2: 4 (23.5%) 3: 1 (5.9%)	<0.001

**Table 2:** Summary of geomean (90% confidence interval) of total and free RPV concentrations, divided by ward. * T_max_ data are reported as the median (range).

	Overall Geomean (CI_90_) N = 34	Cardiac Surgery Geomean (CI_90_) N = 17	Thoracic Surgery Geomean (CI_90_) N = 17
T_max_ * (h)	0.25 (0.08–2)	0.25 (0.083–0.5)	0.25 (0.083–2)
C_max_ total (ng/mL)	1558 (1256–1934)	1120 (816–1680)	2082 (1754–2732)
C_min_ total (ng/mL)	673 (547–827)	445 (369–560)	1022 (776–1277)
AUC_0-3_ total (h * ng/mL)	2975 (2409–3675)	2046 (1638–2642)	4250 (3289–5534)
T_max_ * free (h)	0.25 (0.08–3)	0.25 (0.08–3)	0.25 (0.08–2)
C_max_ free (ng/mL)	97 (73–131)	62 (41–94)	154 (114–208)
C_min_ free (ng/mL)	47 (40–56)	45 (36–58)	50 (37–67)
AUC_0-3_ free (h * ng/mL)	190 (151–239)	148 (109–202)	247 (181–338)

**Table 3:** Coefficients and significance of patients’ anthropometric and clinical characteristics which showed correlations with PK parameters.

	Total Body Weight		BSA		NYHA Score	
	Pearson’s r	*p* Value	Pearson’s r	*p* Value	Pearson’s r	*p* Value
T_max_ total (h)	−0.186	0.291	−0.192	0.276	0.101	0.632
C_max_ total (ng/mL)	−0.526	0.001	−0.547	0.001	0.006	0.979
C_min_ total (ng/mL)	−0.509	0.002	−0.508	0.002	0.176	0.401
Half-life total (h)	0.228	0.194	0.257	0.142	−0.034	0.873
AUC_0–3_ total (h * ng/mL)	−0.566	<0.001	−0.578	<0.001	0.128	0.542
T_max_ free (h)	−0.125	0.481	−0.133	0.454	0.443	0.027
C_max_ free (ng/mL)	−0.531	0.001	−0.567	<0.001	−0.311	0.130
C_min_ free (ng/mL)	−0.476	0.004	−0.499	0.003	0.240	0.248
Half-life free (h)	0.114	0.527	0.094	0.604	0.456	0.022
AUC_0–3_ free (h * ng/mL)	−0.509	0.002	−0.535	0.001	−0.067	0.750

## Data Availability

Data are not available in on-line database due to privacy concerns. Specific anonymized data will be made available upon request.

## Supplementary Materials

The following supporting information can be downloaded at: https://www.mdpi.com/article/10.3390/pharmaceutics18081020/s1, Supplementary Table S1: Summary of the regression coefficients of the univariate linear regression analyses to predict the total RPV peak concentrations; Figure S1: Trend of the predicted total RPV concentrations based on BSA in the study population, with 90% Confidence Intervals. The horizontal dashed line indicates the considered putative MTC, Figure S2: Trend of the predicted total RPV concentrations based on BSA in the study population, with 90% Confidence Intervals. The horizontal dashed line indicates the considered putative MTC; Figure S3: Trend of the predicted total RPV concentrations based on the dose/weight ratio in the study population, with 90% Confidence Intervals. The horizontal dashed line indicates the considered putative MTC.

## Abbreviations

The following abbreviations are used in this manuscript:

LRA	Loco-Regional Anaesthesia
LA	Local Anaesthetics
ESPB	Erector Spinae Plane Block
RPV	Ropivacaine
PK	Pharmacokinetics
PK/PD	Pharmacokinetic/Pharmacodynamic
LAST	Local Anaesthetic Systemic Toxicity
T_max_	Time to reach the maximum concentration
Cmax	Peak plasma concentration
Cmin	Minimum plasma concentration
AUC_0–3_	Area Under the Concentration-Time Curve in the observation period (3 h)
LLOQ	Lower Limit of Quantification
LOD	Limit of Detection
CI_90_	90% Confidence Interval
