# Supplementary material for: Short-Term Pharmacokinetics of Ropivacaine in Arterial Plasma During Erector Spinae Plane Block in Thoracic and Cardiac Surgery
Source: Pharmaceutics. 2026 Aug 17;18(8):1020. doi: 10.3390/pharmaceutics18081020 (PMC13516354; doi:10.3390/pharmaceutics18081020)
Supplement: Supplementary file 1 [file pharmaceutics-18-01020-s001.zip › pharmaceutics-4452183-supplementary.pdf]

Table S1: Summary of the regression coefficients of the univariate linear regression analyses to predict the total RPV peak concentrations.

a

		Unstand. Coeff.		Std. Coeff.			90% confidence interval of B	
Modello		B	Errore standard	Beta	t	Sign.	Low Boundary	High Boundary
		Linear Regression Model from Body Surface Area (BSA)						
1	Constant	6320,966	1149,545		5,499	<.001	3979,420	8662,512
	BSA	-2431,527	658,140	-,547	-3,695	<.001	-3772,114	-1090,941
		Linear Regression Model from Total Body Weight (TBW)						
1	Constant	4474,863	694,040		6,448	<.001	4474,863	694,040
	TBW	-35,733	10,226	-,526	-3,494	,001	-35,733	10,226
		Linear Regression Model from Dose/Weight ratio						
1	Constant	-605,207	654,873		-,924	,362	-1939,140	728,726
	Dose/weight	1140,351	267,785	,601	4,258	<.001	594,890	1685,812

a. Dependent Variable: Cmax_total

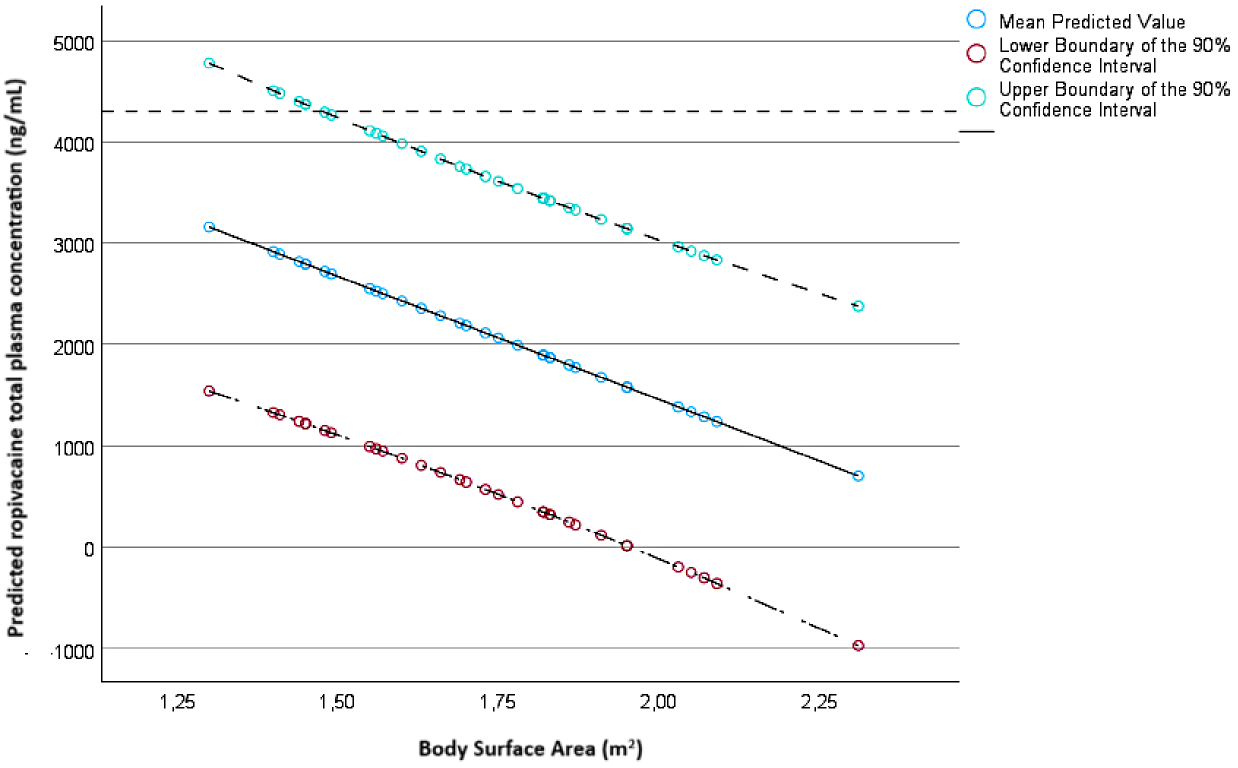

Figure S1: Trend of the predicted total RPV concentrations based on BSA in the study population, with 90% Confidence Intervals. The horizontal dashed line indicates the considered putative MTC.

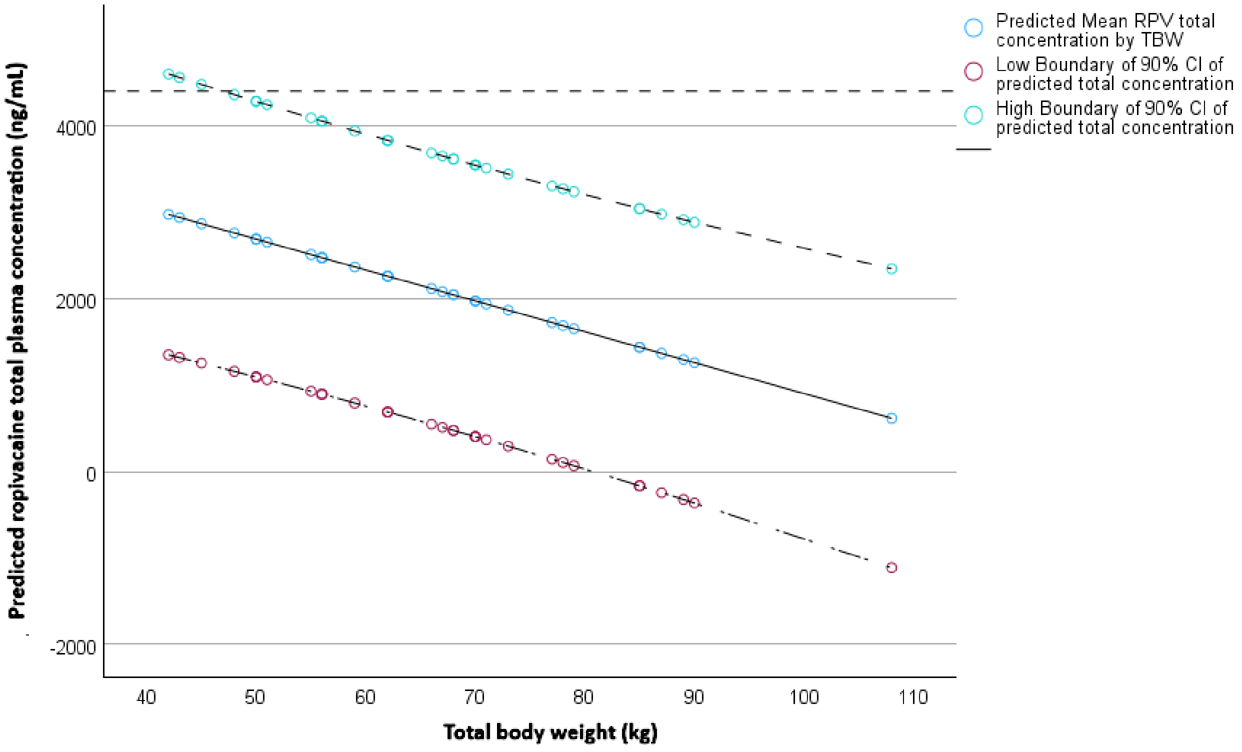

Figure S2: Trend of the predicted total RPV concentrations based on BSA in the study population, with 90% Confidence Intervals. The horizontal dashed line indicates the considered putative MTC.

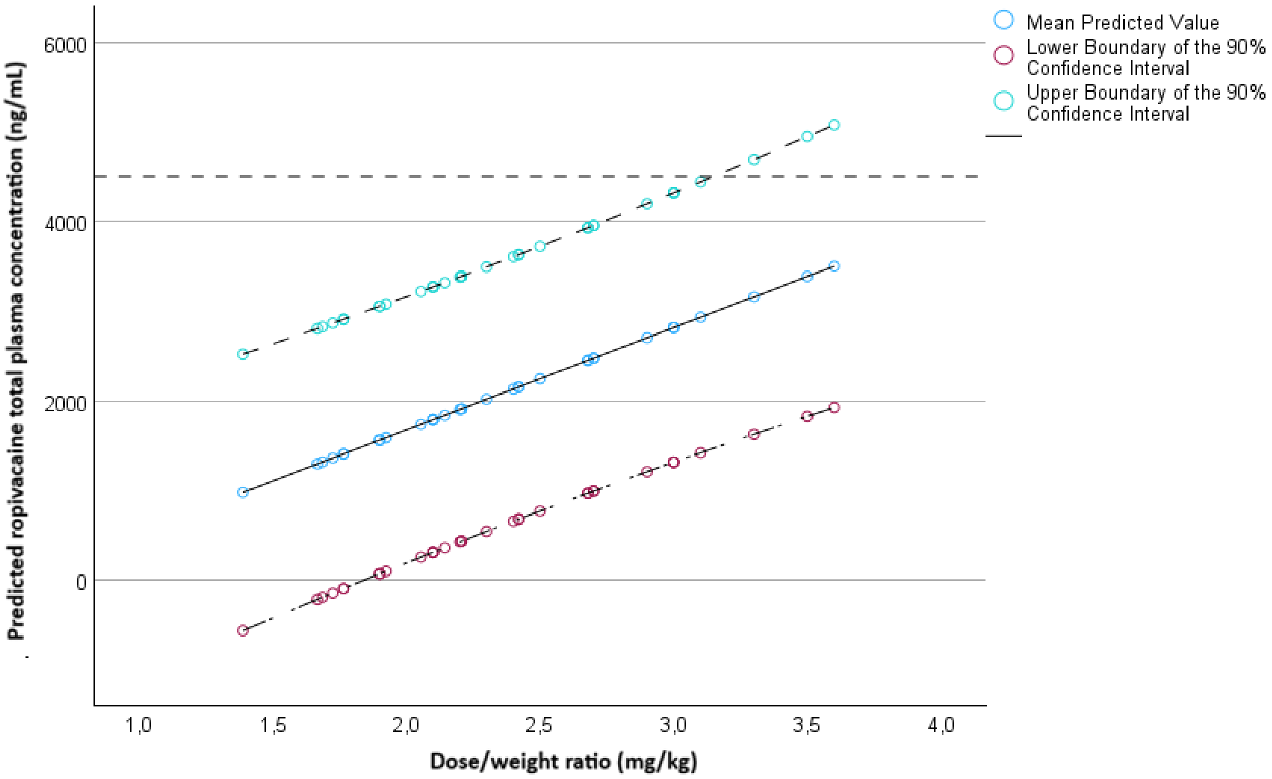

Figure S3: Trend of the predicted total RPV concentrations based on the dose/weight ratio in the study population, with 90% Confidence Intervals. The horizontal dashed line indicates the considered putative MTC.
